# Circular RNA *circFOXO3* promotes prostate cancer progression through sponging *miR‐29a‐3p*

**DOI:** 10.1111/jcmm.14791

**Published:** 2019-11-16

**Authors:** Zhe Kong, Xuechao Wan, Yali Lu, Yingyi Zhang, Yan Huang, Yi Xu, Yajuan Liu, Peiqing Zhao, Xinxin Xiang, Liang Li, Yao Li

**Affiliations:** ^1^ State Key Laboratory of Genetic Engineering Shanghai Engineering Research Center of Industrial Microorganisms School of Life Science Fudan University Shanghai China; ^2^ Department of Oncology Changhai Hospital Second Military Medical University Shanghai China; ^3^ Center of Translational Medicine Central Hospital of Zibo Zibo China; ^4^ Department of Thyroid and Breast Surgery Central Hospital of Zibo Zibo China

**Keywords:** biomarker, *circFOXO3*, *miR‐29a‐3p*, miRNA sponge, prostate cancer (PCa)

## Abstract

Circular RNA *FOXO3* (*CircFOXO3*, also termed as *Hsa_circ_0006404*) is derived from exon 2 of *forkhead box O3* (*FOXO3*) gene, and abnormal expression is shown in different diseases. However, whether *circFOXO3* plays important roles in tumorigenesis and progression of prostate cancer (PCa) remains unclear. In this study, we found that *circFOXO3* was up‐regulated in both PCa tissues and serum samples. Moreover, *circFOXO3* was positively correlated with the Gleason score in PCa samples. *CircFOXO3* was observed to be up‐regulated in Gleason score > 6 PCa samples compared with Gleason score = 6 PCa samples. Knock‐down *circFOXO3* could remarkably inhibit PCa cell cycle, proliferation and promote cell apoptosis in vitro*.* Furthermore, we demonstrated *circFOXO3* could act as *miR‐29a‐3p* sponge to up‐regulate *SLC25A15* expression by bioinformatics analysis, dual‐luciferase reporter assays and biotinylated RNA pull‐down assays. *SLC25A15* could reverse the tumour suppressing roles of knock‐down *circFOXO3* in PCa. Of note, we found that *miR‐29a‐3p* was down‐regulated; however, *SLC25A15* was overexpressed in PCa samples compared with normal tissues. In conclusion, *circFOXO3* acts as a *miR‐29a‐3p* sponge to exhibit oncogenic activity that affects the cell cycle and cell apoptosis in PCa through transcriptional up‐regulation of *SLC25A15*. Our analysis suggests *circFOXO3* could act as promising prostate cancer biomarkers.

## 1 INTRODUCTION

Prostate cancer (PCa) is the disease with the highest number of new cases and the second most common cause of cancer‐related death among American man.^1^, ^2^ Recent years, the incidence and mortality rate of PCa have been significantly increasing in China. Substantial researches have explored the roles of androgen receptor (AR) or other important genes in development and progression of PCa.^3^, ^4^ However, the molecular mechanisms regulating the tumorigenesis and progression of PCa are still unclear.

Recent reports have showed that 80%‐90% RNA molecules are non‐coding RNAs (ncRNAs).^5^ Circular RNAs (circRNAs), a newly emerging endogenous ncRNA, are originated from its parental linear genes by RNA polymerase II and harbour covalently closed circular structure without poly(A) tail and 5′‐3′ polarity.^6^, ^7^ Studies have exhibited that circRNAs are conserved, stable and stage‐/tissue‐specific expression.^8^, ^9^ Recently, scholars paid more attention on circRNAs and identified that lots of circRNAs were significantly differential expression in various cancers, suggested circRNAs might have crucial effect on cancer development.^10^ For example, our group identified *circSMARCA5* was dysregulated and promoted PCa cell growth.^11^ Furthermore, accumulating studies have shown that circRNAs directly combine with miRNAs as ‘miRNA sponges’ and regulate their target genes expression and cancer progression. For example, *circMTO1* suppresses hepatocellular carcinoma progression through regulating p21‐mediated proliferation and invasion by sponging *miR‐9*.^12^

*circFOXO3* is derived from exon 2 of the *FOXO3* gene and contains 1435 nucleotides. Furthermore, Burton Yang *et al* 13 reported that knock‐down of *circFOXO3* promoted cell viability, whereas overexpression of *circFOXO3* inhibited tumour growth and promoted stress‐induced cell apoptosis. The study also demonstrated that *circFOXO3* interacted with MDM2 and decreased MDM2‐induced ubiquitination and degradation of FOXO3, leading to increased FOXO3 protein. Interestingly, other results demonstrated *circFOXO3* could promote protein levels of FOXO3 though interacting with several miRNAs shared with the *FOXO3* linear mRNA.^14^ However, the function and mechanism of *circFOXO3* in PCa remain unclear.

In this study, we discovered the expression of *circFOXO3* was highly expressed in PCa tissue samples and serum samples than controls. Therefore, we knock down *circFOXO3* expression to identify its potential roles and explore possible mechanisms in carcinogenesis of PCa. Here, we demonstrated that *circFOXO3* acted as a *miR‐29a‐3p* sponge to up‐regulated *solute carrier family 25 member 15 (SLC25A15)* and played an oncogenic role in PCa.

## 2 MATERIALS AND METHODS

### 2.1 Tissue samples and serum samples from PCa patients

A total of 53 PCa samples and corresponding adjacent normal prostate tissues were obtained from patients at Fudan University Shanghai Cancer Center. In order to detect the expression levels of *circFOXO3* in serum samples, we collected the serum samples from 26 PCa patient (among 53 PCa patients) and 19 healthy donors (n = 19), who also provided informed consent at Fudan University Shanghai Cancer Center. Those patients did not receive any pre‐operation treatment. The healthy volunteers had no history of cancer until sample accumulation. Samples were centrifuged at 3000 *g* for 10 minutes at 4°C for isolation of serum. The clinicopathological features of the patients are summarized in Table 1 and Table S2. The study was approved by the Research Ethics Committee of Fudan University Shanghai Cancer Center. Informed consent was provided by all patients. All samples were collected and used for gene expression analysis by qRT‐PCR.

**Table 1:** Clinicopathologic characteristics of patient samples and expression of *circFOXO3* in PCa

Characteristics	Number of cases
Age (median = 69.5)	
≤69	26
>69	26
Serum PSA at diagnosis, ng/mL	
<18	23
≥18	25
Median	18
SD	32.34
Mean	28.70
Gleason score	
≤7	28
>7	22
circFOXO3 expression	
≤2.82	26
>2.82	27
Median	2.82
SD	29.54
Mean	49.27

### 2.2 Cell culture

All cell lines (WPMY‐1, LNCaP, 22Rv1, DU145 and PC‐3) were kindly provided by Stem Cell Bank, Chinese Academy of Sciences. WPMY‐1 (the human normal prostate epithelial cell) was expanded in DMEM medium (HyClone), and the PCa cell lines (LNCaP, 22Rv1, DU145 and PC‐3) were maintained in RPMI 1640 medium (HyClone). All of the medium should be added 10% foetal bovine serum before use (FBS, Biological Industries). Then, all cell lines were maintained at 37°C, 5% CO_2_ incubator.

### 2.3 Cell transfection

For knock‐down of *circFOXO3, circFOXO3*‐specific siRNA (si*circFOXO3*) sequence was designed as Burton Yang *et al*’s report^15^ and synthesized by GenePharma. The PCa cells were separately transfected with si*circFOXO3* or negative control (NC) at a final concentration of 50 nmol/L using HilyMax.

For overexpression of miRNA, *hsa‐miR‐143‐3p*, *hsa‐miR‐221‐5p*, *hsa‐miR‐23a* and *hsa‐miR‐29a‐3p* mimics were also designed and synthesized by GenePharma. The PCa cells were separately transfected with miRNA or negative control (NC) at a final concentration of 50 nmol/L using HilyMax. Transfected cells were used for gene expression analysis or other experiments. All the above sequences are shown in Table S1.

### 2.4 RNA isolation and quantitative real‐time‐polymerase chain reaction (qRT‐PCR)

PCa tissue and cultured cells were lysed by MagZol Reagent (Magen), and then, total RNA was extracted. Reverse transcription was performed using PrimeScript™ RT reagent Kit (Takara Bio Inc) according to the manual. qRT‐PCR was conducted in triplicate sample using AceQ qPCR SYBR Green Master Mix (Vazyme Biotech Co Ltd) on LightCycler^®^ 480II (Roche) instrument. The gene expression levels were normalized to the β‐actin. The 2^−ΔΔCt^ method was used for calculating relative expression of genes. Primers used for qRT‐PCR are listed in Table S1.

### 2.5 Cell proliferation assay

Cell proliferation was assessed using the CCK‐8 (Dojindo) as our previous report.^11^ In brief, transfected cells were maintained in 96‐well plates at a density of 5000 cells per well and at 0, 24, 48 and 72 hours post‐treatment, 10 μL CCK‐8 was added to each well and then incubated for 2 hours at 37°C. The optical density was measured at 450 nm by Microplate Reader ELx808 (BioTek).

### 2.6 Flow cytometry analysis of cell cycle

PC‐3, LNCaP‐AI and DU145 cells were maintained in 6‐well plates and transfected with si*circFOXO3* or NC by HilyMax transfection reagents. After 48 hours of transfection, cells were harvested and treated with Triton X‐100 (0.03%) and propidium iodide (PI, 50 ng/mL) for 15 minutes. Cell cycle analysis was performed by FACScalibur flow cytometer (BD).

### 2.7 Annexin V‐FITC apoptosis detection

PC‐3, LNCaP‐AI and DU145 cells were maintained in 6‐well plates and transfected with si*circFOXO3* or NC by HilyMax transfection reagents. At 48 hours after transfection, cells were treated using the FITC‐Annexin V Apoptosis Detection Kit (Dojindo) for 15 minutes at room temperature. The cell apoptosis was measured on FACSCalibur flow cytometer (BD).

### 2.8 Dual‐luciferase reporter assay

A portion of human *SLC25A15* 3’‐UTR (621 bp) and *circFOXO3* including the seed sequence of *miR‐29a‐3p* and *miR‐221‐5p* (570 bp) were separately amplified (Table S1) and inserted into psiCHECK™‐2 firefly/*Renilla* luciferase reporter vector (Promega). Mutagenesis was performed using Mut Express^®^ II Fast Mutagenesis Kit V2 (Vazyme). After transfection for 48 hours, the *Renilla* luciferase activity and firefly luciferase activity were measured by Dual‐Luciferase^®^ Reporter Assay System (Cat. # E1910, Promega).

### 2.9 Biotinylated RNA pull‐down assays

Biotin‐coupled *circFOXO3* probe was designed according to the junction of *circFOXO3* (Table S1). Cellular protein was extracted using lysis buffer [100 mmol/L KCl, 5 mmol/L MgCl_2_, 10 mmol/L HEPES (pH 7.0), 0.5% NP‐40 supplemented with fresh 200 U RNase inhibitor (Yeasen Biotech Co Ltd), 1 mmol/L DTT, 20 mmol/L EDTA, EDTA‐free protease inhibitor cocktail (Roche) and PMSF] and incubated with 3 μg biotin‐coupled probes at 4°C for 2 hours. Then, 30 μL streptavidin‐conjugated magnetic beads (11205D, Invitrogen) were added into the cell lysate and incubated at 4°C for 1 hour. The retrieved RNA was detected by qRT‐PCR assay as described above.

### 2.10 Statistical analysis

All data are reported as the mean ± standard deviation (SD) and represent average of at least three independent experiments. Statistical comparisons between two groups are carried out using Student's two‐tailed unpaired *t* test, and *P* < .05 is considered statistically significant.

## 3 RESULTS

### 3.1 Expression pattern of *circFOXO3* in PCa patients and cell lines

**Figure 1:**
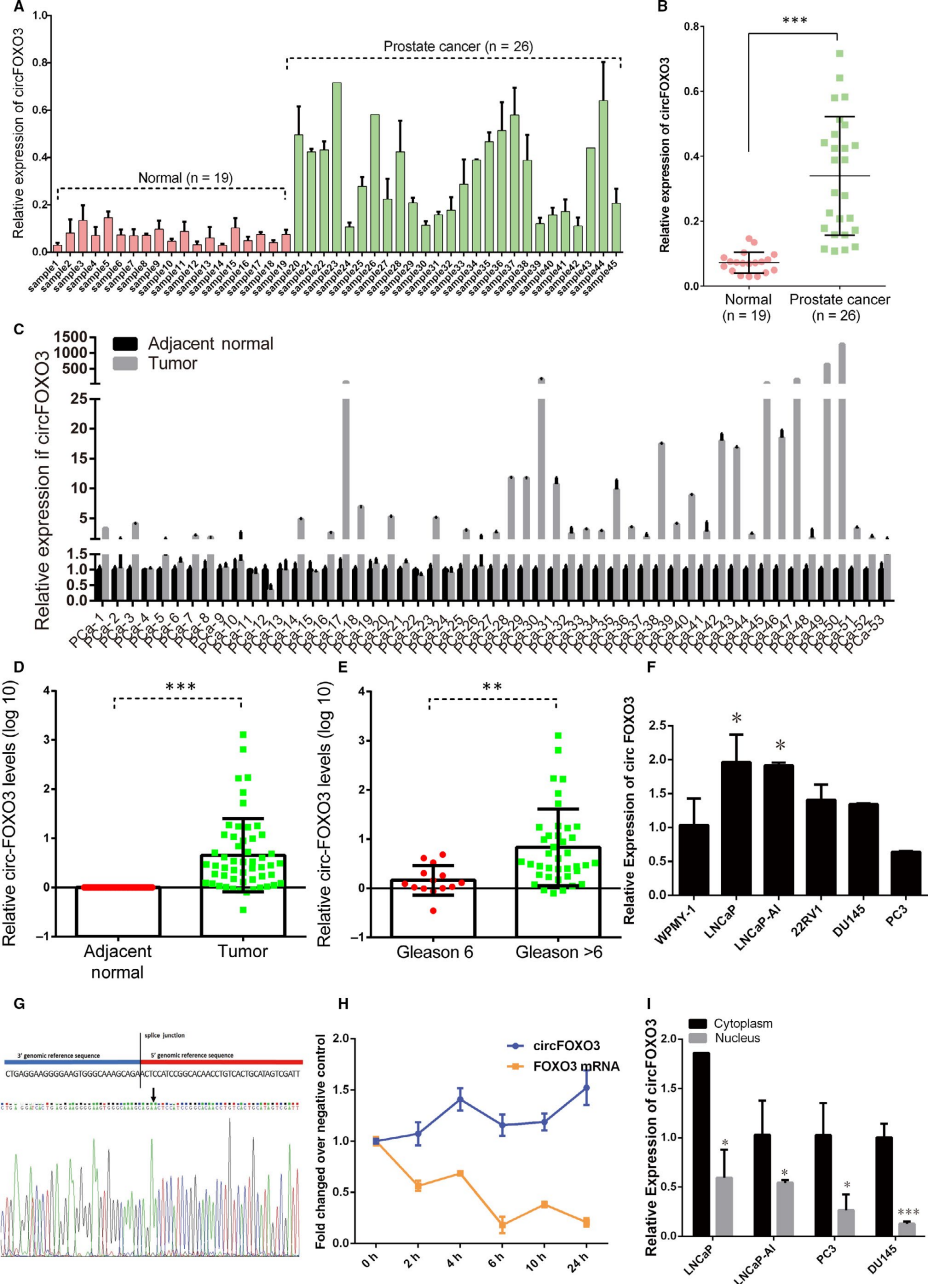
Characteristic and expression of *circFOXO3* in PCa cell lines. (A‐B) qRT‐PCR analysis of *circFOXO3* expression in 26 serum samples from patients with PCa and 19 serum samples from health controls. (C‐D) qRT‐PCR analysis of *circFOXO3* expression in 53 prostatic adenocarcinoma tissue samples and corresponding adjacent normal prostate tissues. E, The expression levels of *circFOXO3* in PCa according to biopsy Gleason scores. F, Relative expression level of *circFOXO3* was measured in normal prostate epithelial cell line WPMY‐1 and five PCa cell lines, LNCaP, LNCaP‐AI, 22Rv1, DU145 and PC‐3 by qRT‐PCR. G, The sequence of *circFOXO3* in circBase (upper part) was consistent with the result of Sanger sequencing (lower part). H, qRT‐PCR analysis of *circFOXO3* and *FOXO3* mRNA after treatment of actinomycin D at the indicated time points in LNCaP cell. I, qRT‐PCR indicated the distribution of *circFOXO3* in nuclear and cytoplasmic fractions of LNCaP, LNCaP‐AI, PC‐3, DU145 cells. Data are presented as the mean ± SD (n = 3). Significance is defined as *P* < .05 (*P < .05; **P < .01; ***P < .001)

*circFOXO3* was reported that it has important roles in diseases, especially in cancers. However, little is known about *circFOXO3* in PCa. Here, we detected *circFOXO3* expression in 26 PCa patient serum samples and 19 health control serum samples using qRT‐PCR. The expression levels of *circFOXO3* in PCa serum samples were significantly higher than those in normal serum samples (Figure 1A‐B). Meanwhile, we investigated *circFOXO3* expression levels in 53 PCa tissue samples and corresponding adjacent normal prostate tissues with qRT‐PCR. Compared with corresponding adjacent normal prostate tissues, *circFOXO3* was also up‐regulated in PCa samples (Figure 1C‐D).

Associations between *circFOXO3* expression levels and the clinicopathologic features of PCa patients are summarized in Table 2. Higher expression levels of *circFOXO3* were significantly correlated with the Gleason score (Figure 1E and Table 2), but were not significantly associated with age and preoperative PSA level (Table 2). *CircFOXO3* was observed to be overexpressed in Gleason score > 6 PCa samples compared with Gleason score = 6 PCa samples. Furthermore, *circFOXO3* expression was detected in LNCaP, LNCaP‐AI, 22Rv1, DU145 and PC‐3 cells by qRT‐PCR, respectively. The result showed that *circFOXO3* was up‐regulated in PCa cells compared with WPMY‐1 cells, thereby confirming that *circFOXO3* was overexpressed in PCa (Figure 1F). Those results raise the possibility that the dysregulated *circFOXO3* might play an important role in PCa progression.

**Table 2:** Correlation between circFOXO3 expression and clinicopathologic features in PCa patients

Features	CircFOXO3 expression		*P*‐value
	Low	High	
Age at surgery (n = 52)			
Median (range)	69.88 (68.29‐71.47)	67.96 (66.67‐69.25)	.352
Mean	69.88	67.96	
Pre‐operation PSA level (ng/mL) (n = 48)			
Median (range)	21.19 (16.41, 25.97)	36.86 (28.85, 44.88)	.1017
Mean	21.19	36.86	
Gleason score (n = 50)***			
Gleason < 7	12	2	.0004
Gleason ≥ 7	13	23	

*** *P* < .01.

The expression of *circFOXO3* was quantified by qRT‐PCR with divergent primers (Table S1), and the distinct product was further confirmed by Sanger sequencing (Figure 1G). The stability and localization of *circFOXO3* were explored in PCa cells. We detected the expression of *circFOXO3* and *FOXO3* at 0, 2, 4, 8, 10 and 24 hours after treatment with actinomycin D, a transcription inhibitor, by qRT‐PCR. The result showed that *circFOXO3* was much more stable than *FOXO3* mRNA (Figure 1H). Furthermore, we performed subcellular fractionation and detected the cellular localization of *circFOXO3* by qRT‐PCR in LNCaP, LNCaP‐AI, PC‐3 and DU145 cells. The result revealed that *circFOXO3* was predominantly cytoplasmic (Figure 1I). Taken together, our results show that *circFOXO3* is a stable, predominantly cytoplasmic circRNA and up‐regulated in PCa.

### 3.2 Knock‐down of *circFOXO3* inhibited proliferation and promoted apoptosis of PCa cells

**Figure 2:**
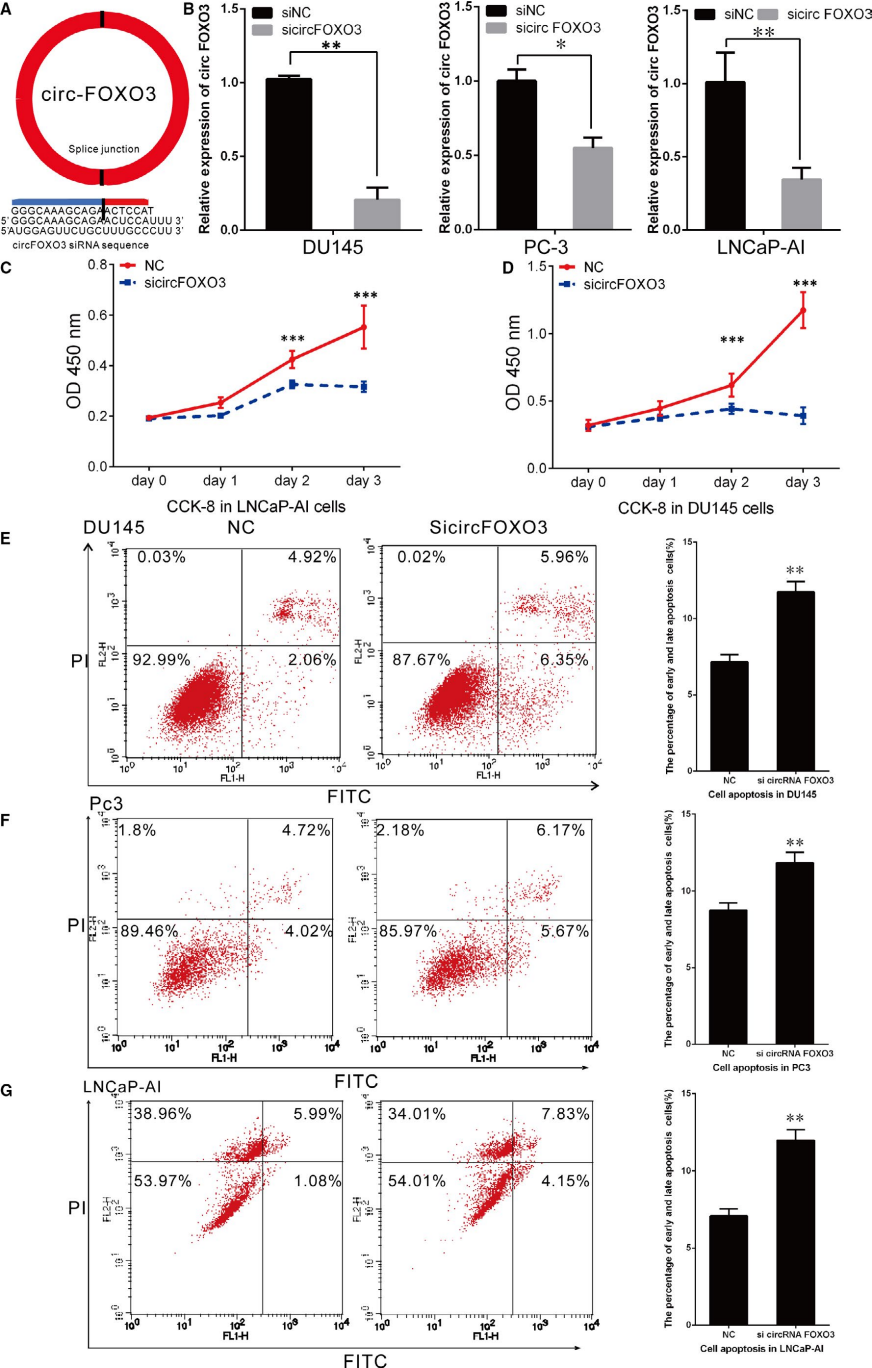
*CircFOXO3* silencing suppressed cell proliferation but promoted cell apoptosis in PCa cell lines. A, A siRNA was designed to specifically target *circFOXO3*. B, The efficiency of si*circFOXO3* was confirmed by qRT‐PCR. (C‐D) Cell proliferation analysis was performed with CCK‐8 assay in LNCaP‐AI, DU145. Cells transfected with si*circFOXO3* and NC were seeded into 96‐well plate at 5000 cells/well and examined at time points of 0, 24, 48 and 72 h. Knock‐down of *circFOXO3* inhibited cell proliferation in LNCaP‐AI and DU145. (E‐G) Cell apoptosis assay was performed in DU145, PC‐3 and LNCaP‐AI cells. Cells were transfected with NC or si*circFOXO3*, and stained with PI and FITC. Knock‐down of *circFOXO3* increased the percentage of cell apoptosis. Data are presented as the mean ± SD (n = 3). Significance is defined as *P* < .05 (*P < .05; **P < .01; ***P < .001)

To investigate the biological roles of *circFOXO3* in PCa, we performed loss‐of‐function experiments in PCa cell lines. First, we used siRNA reported in Burton Yang *et al*’s study specifically targeting *circFOXO3* to knock down its expression.^15^ Compared to linear RNA, back‐splicing site was the circRNA‐specific sequence. Si*circFOXO3* was designed to target the back‐splicing site (Figure 2A). Compared to treatment with NC, si*circFOXO3* substantially decreased *circFOXO3* levels in LNCaP‐AI, PC‐3 and DU145 cells (Figure 2B).

Cell proliferation assay showed that knock‐down of *circFOXO3* could suppressed cell proliferation compared with the cells transfected with the NC in LNCaP‐AI, suggesting endogenous *circFOXO3* might be involved in the cell proliferation of PCa (Figure 2C). In DU145 cells, we also found *circFOXO3* silencing suppressed cell proliferation compared with the NC (Figure 2D).

We next investigated the effect of *circFOXO3* in cell apoptosis of PCa. DU145, PC‐3 and LNCaP‐AI cells were separately treated with si*circFOXO3* or NC, and at 48 hours after transfection, we analysed cell apoptosis by flow cytometry. The apoptosis rates of the si*circFOXO3* and NC were 11.74 ± 0.68% and 7.16 ± 0.48% in DU145 cells, 11.55 ± 0.27% and 8.86 ± 0.16% in PC‐3 cells, and 12.18 ± 0.22% and 6.94 ± 0.32% in LNCaP‐AI, respectively (Figure 2E‐G). The result indicates that knock‐down of *circFOXO3* expression could significantly promote apoptosis of PCa cells.

### 3.3 *circFOXO3* knock‐down affected cell cycle progression of PCa cells

**Figure 3:**
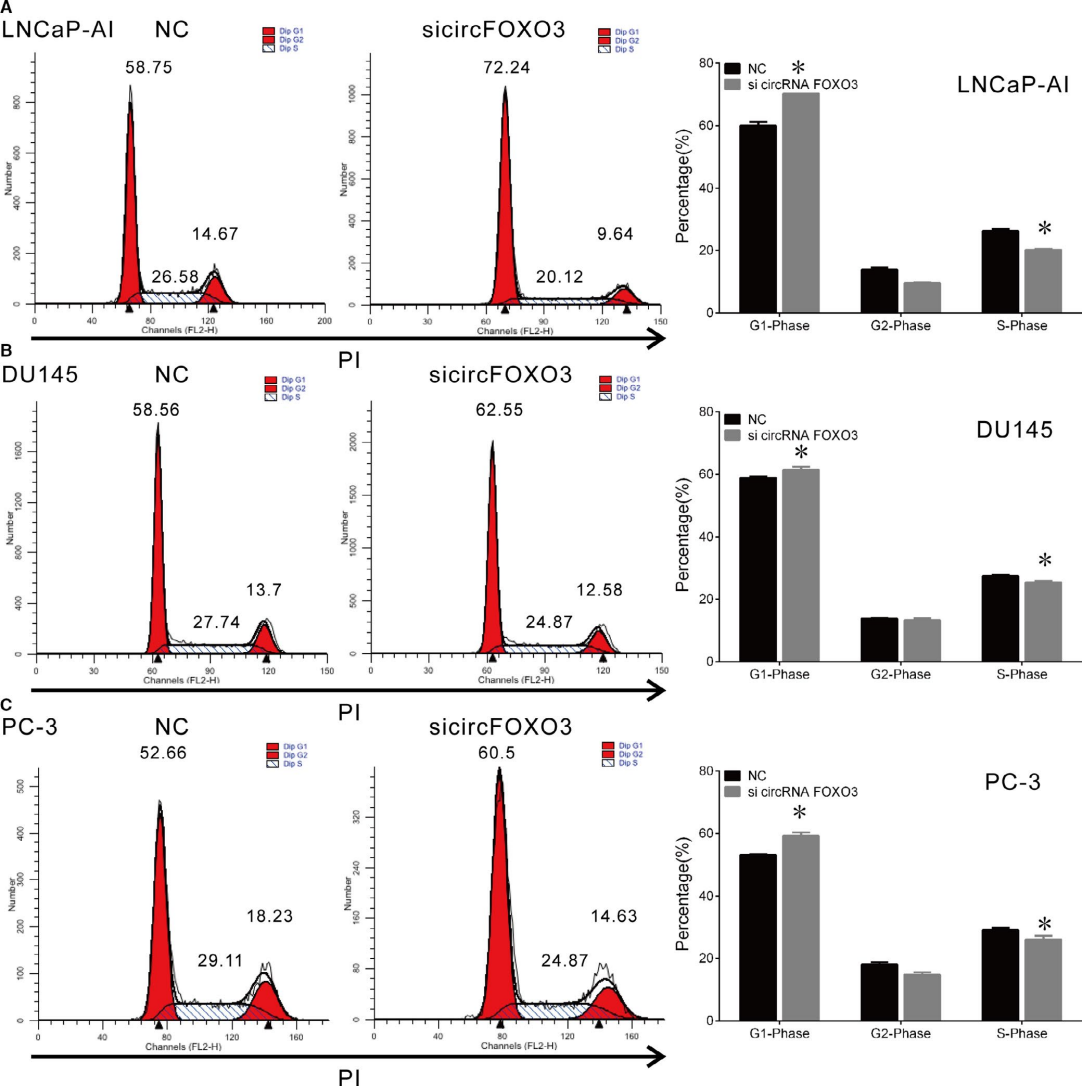
*CircFOXO3* knock‐down affects cell cycle progression in PCa cell lines. (A‐C) Cell cycle assay was performed in LNCaP‐AI, DU145 and PC‐3 cells. Cells were transfected with si*circFOXO3* or NC for 48 h, stained with PI and evaluated with a FACScalibur flow cytometer. Knock‐down of *circFOXO3* increased the number of cells in G1 phase and decreased the number of cells in S and G2 phases. Data are presented as the mean ± SD (n = 3). Significance is defined as *P* < .05 (*P < .05)

Next, we detected the effect of *circFOXO3* on the PCa cell cycle progression using flow cytometry. Knock‐down of *circFOXO3* increased the percentage of cells in G0/G1 phase (58.75% to 72.24% in LNCaP‐AI, 58.56% to 62.55% in DU145 and 52.66% to 60.5% in PC‐3 cells) and decreased the percentage of S phase (26.58% to 20.12% in LNCaP‐AI, 27.74% to 24.87% in DU145 and 29.11% to 24.87% in PC‐3 cells) (*P* < .05; Figure 3). Taken together, these results indicate that the effect of silencing *circFOXO3* on PCa cell proliferation could be attributed to its promotion of apoptosis and cell cycle arrest.

### 3.4 Identification of *circFOXO3* mediated ceRNA and confirmation of the sponging effect between *circFOXO3* and *miR‐29a‐3p*

**Figure 4:**
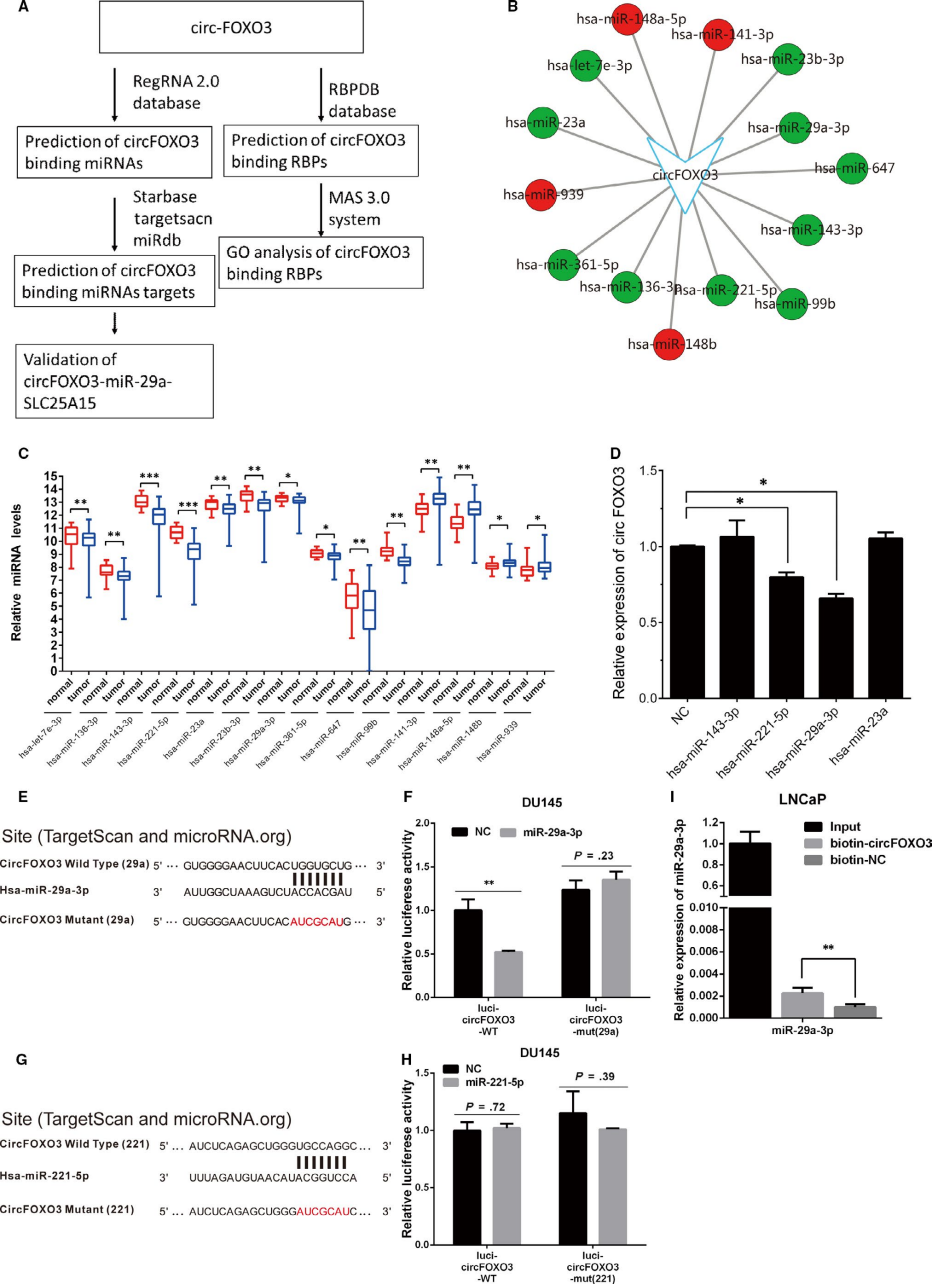
*CircFOXO3* acted as a *miR‐29a‐3p* sponge. A, Bioinformatics methods paragraph to describe the procedure of bioinformatics analysis. B, Intersections of *circFOXO3* targeted miRNA. The red and green circles separately indicated up‐ and down‐regulated miRNAs. C, Fourteen miRNAs were differently expressed in PCa samples using http://www.ncbi.nlm.nih.gov/geo/query/acc.cgi?acc=GSE21036. D, qRT‐PCR analysis of *circFOXO3* expression transfected with four tumour‐suppressive miRNAs (including *hsa‐miR‐143‐3p, hsa‐miR‐23a, hsa‐miR‐29a‐3p and hsa‐miR‐221‐5p*). E, Schematic illustration indicating the wild‐type or mutant target site of *circFOXO3* and base pairing of *miR‐29a‐3p*. F, Luciferase assay in DU145 cells. Overexpression of *miR‐29a‐3p* decreased the luciferase activity of *circFOXO3*, while *miR‐29a‐3p* had no significant effect on luciferase activity of *circFOXO3* with the mutated target site. G, Schematic illustration indicating the wild‐type or mutant target site of *circFOXO3* and base pairing of *miR‐221‐5p*. H, Luciferase assay in DU145 cells. Overexpression of *miR‐221‐5p* had no significant effect on luciferase activity of *circFOXO3* with wild‐type or mutated target site. I, *miR‐29a‐3p* was pulled down and enriched with *circFOXO3* probe and then detected by qRT‐PCR. Data are presented as the mean ± SD (n = 3). Significance is defined as *P* < .05 (*P < .05; **P < .01; ***P < .001)

As mentioned in previous reports, natural circRNAs could function as efficient microRNA sponges to regulate protein‐coding genes.^12^ To explore potential mechanism of *circFOXO3* regulating PCa progression, we constructed *circFOXO3*‐mediated competing endogenous RNA (ceRNA) networks. By using RegRNA 2.0 database, we identified 14 miRNAs targeted to *circFOXO3* (Figure 4A‐B).

Except as RNA sponge, mounting evidence suggested circRNAs could sequester proteins.^16^ Here, we constructed a *circFOXO3* interaction protein network. By using RBPDB database, eight RNA binding proteins (MBNL1, NONO, SFRS9, RBM4, SFRS1, FUS, EIF4B and RBMX) with relative score ≥ 0.99 were identified to interact with *circFOXO3* (Figure S1A). Next, we analysed the interaction proteins of these RNA binding proteins got from NCBI interaction protein database. As shown in Figure S1A, the circRNA‐related interaction protein network contained 481 nodes and 674 edges. Bioinformatics analysis showed *circFOXO3*‐related network was mainly associated with regulating RNA splicing (including RNA splicing, nuclear mRNA splicing, mRNA processing, mRNA transport and mRNA catabolism) and cell proliferation (including cell cycle, anti‐apoptosis, DNA replication, cell cycle arrest, DNA repair and mitosis) (Figure S1B).

To test the hypothesis that *circFOXO3* promoted PCa proliferation by regulating these miRNAs, we analysed these miRNA expression patterns in public data http://www.ncbi.nlm.nih.gov/geo/query/acc.cgi?acc=GSE21036,^17^ which contained 28 corresponding adjacent normal prostate tissues and 113 PCa samples. Our results showed 10 of 14 miRNAs (including *hsa‐let‐7e‐3p, hsa‐miR‐136‐3p, hsa‐miR‐143‐3p, hsa‐miR‐221‐5p, hsa‐miR‐23a, hsa‐miR‐23b‐3p, hsa‐miR‐29a‐3p, hsa‐miR‐361‐5p, hsa‐miR‐647 and hsa‐miR‐99b*) were down‐regulated, and only 4 miRNAs (including *hsa‐miR‐141‐3p, hsa‐miR‐148a‐5p, hsa‐miR‐148b and hsa‐miR‐939*) were up‐regulated in PCa (Figure 4B‐C). These results are consistent with our conclusion that *circFOXO3* may act as an oncogenic circRNA in PCa.

In order to validate our ceRNA network analysis, we selected three of the most significantly down‐regulated miRNAs (including *hsa‐miR‐143‐3p, hsa‐miR‐221‐5p and hsa‐miR‐23a*) and *hsa‐miR‐29a‐3p* identified tumour‐suppressive miRNAs in PCa for further study. In our previous reports, we found *hsa‐miR‐29a‐3p* inhibited proliferation and induced apoptosis of PCa cells.^18^ Furthermore, we detected *circFOXO3* expression level after overexpression of *hsa‐miR‐143‐3p, hsa‐miR‐221‐5p*, *hsa‐miR‐29a‐3p and hsa‐miR‐23a* in PC‐3 cells. The result showed that only overexpression of *hsa‐miR‐29a‐3p* or *hsa‐miR‐221‐5p* inhibited *circFOXO3* expression level (Figure 4D), which suggested that *circFOXO3* might interacted with *hsa‐miR‐29a‐3p* and *hsa‐miR‐221‐5p*.

To validate the targeting relationship between *hsa‐miR‐29a‐3p* and *circFOXO3*, we performed dual‐luciferase assay. Bioinformatics prediction showed the predicted *miR‐29a‐3p* binding site in *circFOXO3*, and then, we constructed psiCHECK^™^‐2 luciferase reporter plasmid, containing the fragment of *circFOXO3* with the predicted *hsa‐miR‐29a‐3p* binding site [*circFOXO3* Wild Type (29a)] or mutant *hsa‐miR‐29a‐3p* target site [*circFOXO3* Mutant (29a)] (Figure 4E). Then, we transfected both *miR‐29a‐3p* mimic and luciferase reporter plasmid into DU145 cells. Compared with miR‐NC, *miR‐29a‐3p* significantly reduced the luciferase activity of *circFOXO3* at ~ 50%, while *miR‐29a‐3p* had no significant effect on luciferase activity of *circFOXO3* with the mutated target site (Figure 4F). Similarly, we also detected whether *miR‐221‐5p* directly interacted with *circFOXO3* by dual‐luciferase reported assays. The result showed that *miR‐221‐5p* did not affect the luciferase reporter activity of *ircFOXO3* with either wild‐type or mutated target site (Figure 4G‐H). Taken together, *circFOXO3* directly interacted with *miR‐29a‐3p* but not *miR‐221‐5p*. Furthermore, biotin‐coupled *circFOXO3* probe pull‐down assay showed that *miR‐29a‐3p* was detected in the *circFOXO3* pull‐down pellet compared with NC group (Figure 4I). These results indicate that *circFOXO3* acts as a sponge for *miR‐29a‐3p*.

### 3.5 *circFOXO3* regulates *miR‐29a‐3p* target expression

**Figure 5:**
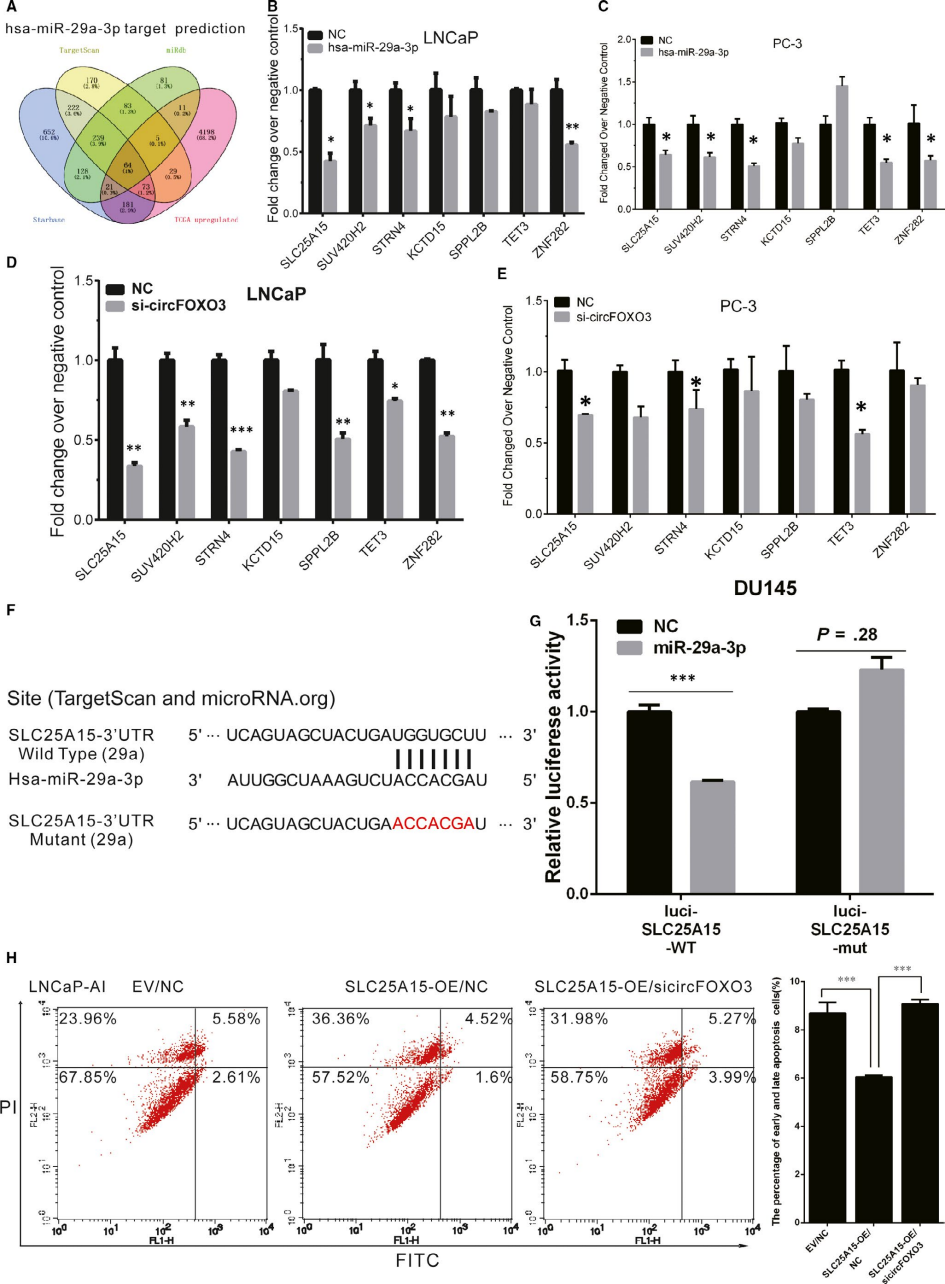
*CircFOXO3/miR‐29a‐3p* cascade regulated *SLC25A15* expression. A, Venn diagrams for *miR‐29a‐3p* targeted genes using three open‐access database, including TargetScan, miRDB and starBase. A total of 64 candidate targets of *miR‐29a‐3p* were identified to be up‐regulated in PCa samples using TCGA data set. (B‐C) qRT‐PCR analysis expression levels of 7 genes (including *SLC25A15, SUV420H2, STRN4, KCTD15, SPPL2B, TET3 and ZNF282*) selected as ceRNA targets of *circFOXO3/miR‐29a‐3p* cascade after overexpressing *miR‐29a‐3p* in DU145 (B) and PC‐3 (C) cells. (D‐E) qRT‐PCR analysis of 7 genes (including *SLC25A15, SUV420H2, STRN4, KCTD15, SPPL2B, TET3* and *ZNF282*) selected as ceRNA targets of *circFOXO3/miR‐29a‐3p* cascade after silencing of *circFOXO3* in DU145 (D) and PC‐3 (E) cells. F, Schematic illustration indicating the wild‐type or mutant 3’UTR of *SLC25A15* and base pairing of *miR‐29a‐3p*. G, Luciferase assay in DU145 cells. Overexpressing *miR‐29a‐3p* decreased the luciferase activity of *SLC25A15*. H, Cell apoptosis assay was performed in LNCaP‐AI cell. Overexpressing *SLC25A15* significantly suppressed cell apoptosis; however, this effect was significantly abrogated by co‐transfection with si*circFOXO3*. Data are presented as the mean ± SD (n = 3). Significance is defined as *P* < .05 (*P < .05; **P < .01; ***P < .001)

To identify novel downstream targets of *miR‐29a‐3p,* we first conducted bioinformatics analysis by using TargetScan (http://www.targetscan.org), miRDB (mirdb.org) and starBase v2.0 (starbase.sysu.edu.cn). A total of 64 candidate targets of *miR‐29a‐3p* were identified to be overexpressed in PCa samples using TCGA data set (Figure 5A). In the present study, 7 genes (including *SLC25A15*, *SUV420H2*, *STRN4*, *KCTD15*, *SPPL2B*, *TET3* and *ZNF282*) were selected as ceRNA targets of *circFOXO3/miR‐29a‐3p* cascade. Next, we separately overexpressed *miR‐29a‐3p* in DU145, PC‐3 cells (Figure 5B‐C) and LNCaP, 22Rv1 cells (Figure S1C‐D), and observed *SLC25A15*, *SUV420H2*, *STRN4* and ZNF282 were significantly suppressed by *miR‐29a‐3p* in PCa cell lines. Interestingly, knock‐down of *circFOXO3* could also inhibit *SLC25A15, STRN4* and *TET3* expression (Figure 5D‐E and Figure S1E‐F). Hence, we consider *SLC25A15* is a direct target of *circFOXO3*/*miR‐29a‐3p*.

To further confirm whether *SLC25A15* is a direct target gene of *miR‐29a‐3p, SLC25A15*‐3′UTR with *miR‐29a‐3p* wild‐type or mutant binding site was separately cloned into downstream of the luciferase reporter gene (Figure 5F). Luciferase reporter assay results showed *miR‐29a‐3p* mimics significantly decreased the luciferase activity of *SLC25A15*‐3’UTR vectors compared with miR‐NC, while *miR‐29a‐3p* mimics had no significant effect on luciferase activity *SLC25A15*‐3’UTR with the mutated target site (Figure 5G). Furthermore, apoptotic results showed that overexpression of *SLC25A15* significantly suppressed apoptosis in LNCaP‐AI cell; however, this effect was significantly abrogated by co‐transfection with si*circFOXO3* (Figure 5H). These results indicate *circFOXO3* acts as a *miR‐29a‐3p* sponge to regulate *SLC25A15* expression.

### 3.6 The expression of *miR‐29a‐3p* was down‐regulated in PCa

**Figure 6:**
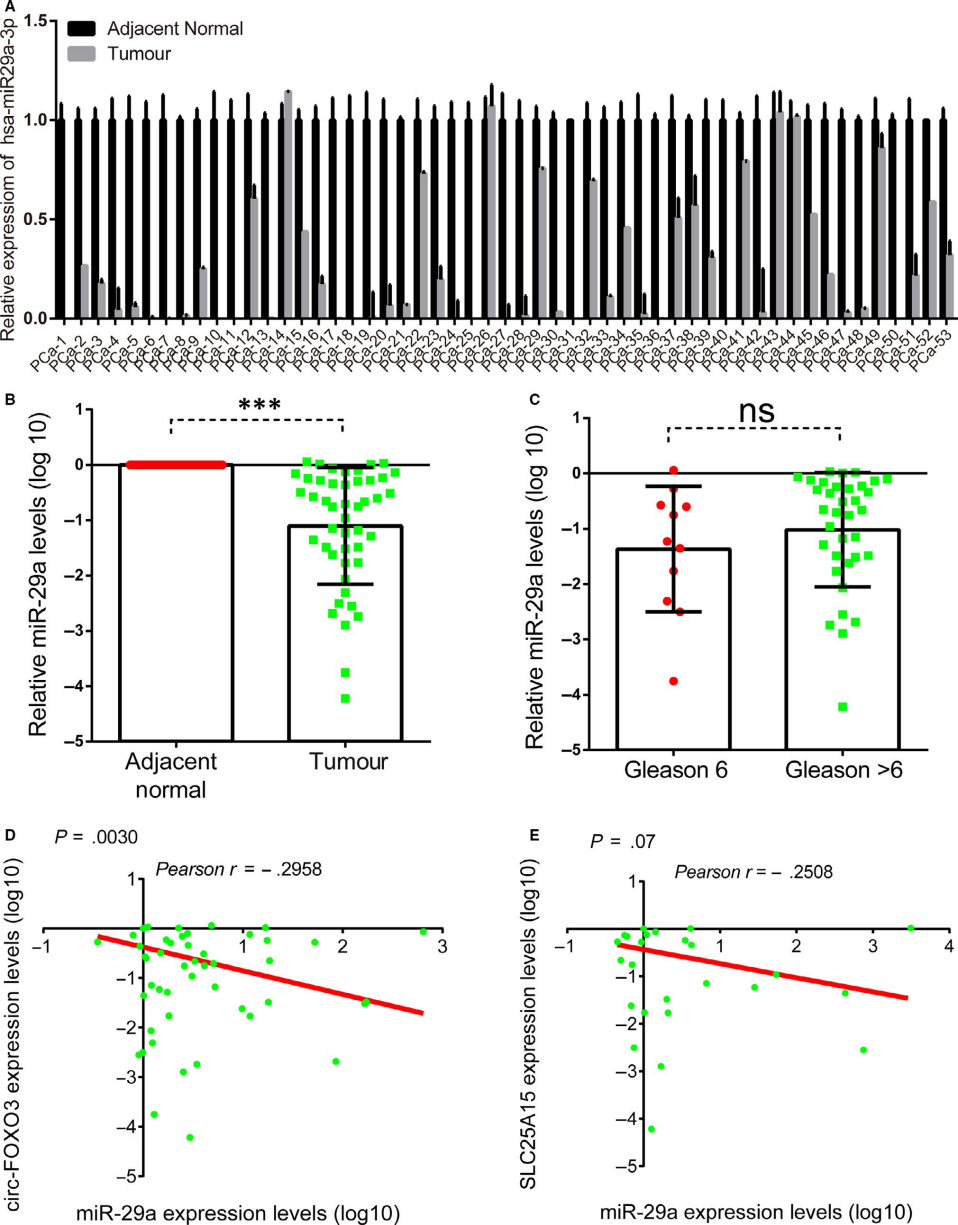
The expression of *miR‐29a‐3p* was negatively correlated with *circFOXO3* and *SLC25A15*. (A‐B) qRT‐PCR analysis of *miR‐29a‐3p* expression in 53 prostatic adenocarcinoma tissue samples and corresponding adjacent normal prostate tissues. C, The expression levels of *circFOXO3* in PCa according to biopsy Gleason scores. (D‐E) The Pearson correlation coefficient analyses of *miR‐29a‐3p* and *circFOXO3* (D), *SLC25A15* (E). Data are presented as the mean ± SD (n = 3). Significance is defined as *P* < .05 (***P < .001)

Our previous studies had showed that *miR‐29a‐3p* played as a tumour suppressor in PCa.^18^ However, the expression pattern of *miR‐29a‐3p* in PCa remained unclear. Therefore, we detected the expression of *miR‐29a‐3p* in PCa tissue. The result showed that *miR‐29a‐3p* was lowly expressed in PCa tissue sample compared with corresponding adjacent normal prostate tissues (Figure 6A‐B). However, we did not observe a significant correlation between the *miR‐29a‐3p* expression and Gleason score, which may be due to the limited sample size (Figure 6C). Very interestingly, we observed a significantly negative correlation between *miR‐29a‐3p* and *circFOXO3* or *SLC25A15* (Figure 6D‐E), suggested the existence of *circFOXO3*/*miR‐29a‐3p/SLC25A15* in PCa samples.

### 3.7 *SLC25A15* was up‐regulated in PCa tissues

**Figure 7:**
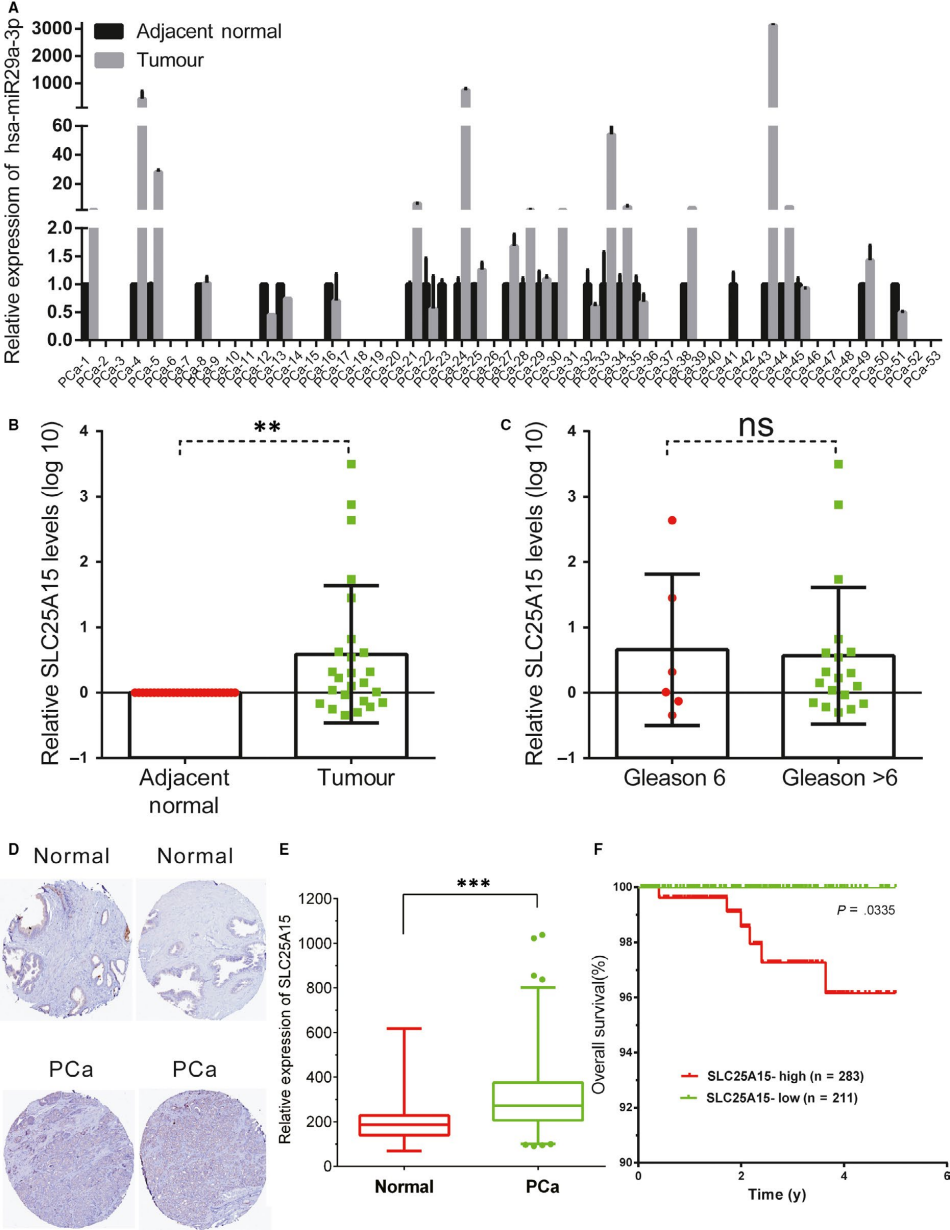
The expression of *SLC25A15* in PCa. (A‐B) qRT‐PCR analysis of *SLC25A15* mRNA expression level in 53 prostatic adenocarcinoma tissue samples and corresponding adjacent normal prostate tissues. C, The expression levels of *circFOXO3* in PCa according to biopsy Gleason scores. D, *SLC25A15* protein levels were overexpressed in PCa samples compared with normal samples by analysing Human Protein Atlas. E, TCGA data set analysis showed *SLC25A15* mRNA levels were overexpressed in PCa samples. F, Kaplan‐Meier curves for survival time in patients with PCa according to expression of *SLC25A15*. Significance was defined as *P* < .05 (**P < .01; ***P < .001)

We compared *SLC25A15* expression levels in 53 PCa tissue samples and corresponding adjacent normal prostate tissues by qRT‐PCR. Our analysis revealed that *SLC25A15* mRNA expression was up‐regulated in PCa samples compared with corresponding adjacent normal prostate tissues (Figure 7A‐B). However, we did not observe a significant correlation between the *SLC25A15* expression and Gleason score (Figure 7C). To further compare *SLC25A15* protein levels in PCa and normal tissues, we analysed *SLC25A15* expression in Human Protein Atlas (https://www.proteinatlas.org/). We also observed *SLC25A15* was overexpressed in PCa samples compared with normal samples (Figure 7D). More importantly, TCGA data set analysis revealed *SLC25A15* was up‐regulated in PCa samples (Figure 7E) and increased *SLC25A15* expression in PCa tissues was significantly correlated with shorter 5‐year overall survival time of PCa patients, shown as Kaplan‐Meier survival curve (Figure 7F). Together, these data suggest that *SLC25A15* is up‐regulated in PCa tissues.

## 4 DISCUSSION

Circular RNAs (circRNAs), a covalently closed circular structural RNA, are originated from parental linear genes by RNA polymerase II and synthesized by alternative splicing. Numerous circRNAs may act as diagnostic and therapeutic biomarkers for various diseases due to their high biological stability. Recently, reports have shown that differently expressed circRNAs in blood could act as diagnostic biomarkers. Zhao *et al* 19 reported that *hsa_circ_0054633* in blood acted as a novel biomarker of pre‐diabetes and type 2 diabetes mellitus. Guo *et al* 20 also identified *hsa_circ_0000190* in blood may be a novel non‐invasive biomarker for the diagnosis of gastric cancer. Here, we detected the expression of *circFOXO3* in PCa serum samples. The results showed the levels of *circFOXO3* expression in PCa serum samples were significantly higher than those in normal samples, which suggested *circFOXO3* could acted as a diagnostic biomarker of PCa. Of note, our results for the first time demonstrated that *circFOXO3* was overexpressed in tumour tissues and positively correlated with Gleason score in PCa. The cellular localization determines the potential function of a gene. Here, RNA fractionation analyses revealed that *circFOXO3* was predominantly cytoplasmic in PCa cells.

Accumulating studies showed that *circFOXO3* was involved in progression of diseases, such as cardiac senescence^21^ and non–small‐cell lung cancer.^22^ Moreover, Burton Yang *et al* reported *circFOXO3* interacted with p21 and CDK2, and retarded cell cycle progression.^15^ They also detected that *circFOXO3* induced tumour apoptosis though enhancing FOXO3 activity.^13^ However, whether *circFOXO3* plays a role in PCa remains unclear. Therefore, we knock down *circFOXO3* and then explored the potential biological function in PCa development. Intriguingly, our data are not consistent with previous studies. In this study, we found *circFOXO3* silencing significantly inhibited the growth of PCa cells by affecting cell cycle progression (LNCaP‐AI and DU145 cells) and cell apoptosis. These results suggested that *circFOXO3* played an oncogenic role in the development of PCa. Importantly, this is the first study to investigate the molecular function and mechanism of *circFOXO3* in PCa.

To explore the potential mechanisms of *circFOXO3* regulating PCa progression, we constructed a circRNA‐mediated ceRNA network in PCa by using bioinformatics analysis. Our results showed *circFOXO3* may act as a miRNA sponge to bind miRNAs. Interestingly, more than 71 per cent of these miRNAs (including *hsa‐let‐7e‐3p, hsa‐miR‐136‐3p, hsa‐miR‐143‐3p, hsa‐miR‐221‐5p, hsa‐miR‐23a, hsa‐miR‐23b‐3p, hsa‐miR‐29a‐3p, hsa‐miR‐361‐5p, hsa‐miR‐647 and hsa‐miR‐99b*) were down‐regulated and only 4 miRNAs (including *hsa‐miR‐141‐3p, hsa‐miR‐148a‐5p, hsa‐miR‐148b and hsa‐miR‐939*) were up‐regulated in PCa. Of note, previous reports had shown that *hsa‐miR‐143‐3p*,^23^ *hsa‐miR‐221‐3p*,^24^ *hsa‐miR‐23b‐3p*,^25^ *hsa‐miR‐29a‐3p* 18 and *hsa‐miR‐361‐5p* 26 acted as tumour‐suppressive roles in PCa. Furthermore, we performed dual‐luciferase assay and biotinylated RNA pull‐down assay and validated *circFOXO3* acted as a *miR‐29a‐3p* sponge to regulate its target genes and progression of PCa. In addition, *MiR‐29a‐3p* was reported as a tumour suppressor in PCa.^27^, ^28^ In our previous study, we also found *hsa‐miR‐29a‐3p* suppressed cell proliferation and induced apoptosis in PCa.^18^ We further validated *SLC25A15* was the target of *miR‐29a‐3p*. Therefore, we investigated if, in our system, *circFOXO3* played significant roles in PCa development through *circFOXO3*/*miR‐29a‐3p*/*SLC25A15* axis. Our data showed that overexpression of *SLC25A15* significantly suppressed apoptosis; however, this phenomenon was remarkably abrogated by co‐transfection with si*circFOXO3.* By detecting *circFOXO3*/*miR‐29a‐3p*/*SLC25A15* axis expression in PCa samples, our results also showed a significantly negative correlation between *miR‐29a‐3p* and *circFOXO3* or *SLC25A15* in PCa tissues. These results could explain, at least in part, how *circFOXO3* promotes PCa progression. Very interestingly, *miR‐29a‐3p* belonged to *miR‐29* family and shared the similar seed region with *miR‐29b/c*. Despite this study focused on the effect of *circFOXO3* on *miR‐29a*, we could reasonably hypothesize that *circFOXO3* could also sponge *miR‐29b/c*. *miR‐29b* is a miRNA that regulates both osteoblastogenesis and osteoclastogenesis.^29^ Furthermore, serum *miR‐29b* is down‐regulated in PCa patients and this increases the formation of osteolytic lesions, influencing osteoblast and osteoclast differentiation and activities.^30^ These results suggested that overexpression in serum of *circFOXO3* could influence osteolytic lesions formation through these processes in PCa. However, the further validation is still needed.

*SLC25A15* transports ornithine across the inner membrane of mitochondria from the cytosol to the mitochondrial matrix and plays an important role in regulating the urea cycle.^31^ However, the roles of *SLC25A15* in cancers remain largely unclear. In this study, we found *SLC25A15* was a downstream target of *circFOXO3* and up‐regulated in PCa samples compared with corresponding adjacent normal prostate tissues. Public data sets analysis also showed *SLC25A15* mRNA, and protein levels were overexpressed in PCa samples. More important, we found highly expressed *SLC25A15* in PCa tissues was significantly associated with poor prognosis in patients with PCa. We consider this study provides useful information for exploring potential therapeutic and prognostic targets for PCa intervention.

## CONFLICT OF INTEREST

The authors declare no competing interests.

## AUTHORS’ CONTRIBUTIONS

Zhe Kong, Xuechao Wan, Yali Lu, Liang Li and Yao Li conceived and designed the study; Zhe Kong, Xuechao Wan and Yali Lu developed the methodology; Zhe Kong, Xuechao Wan, Yingyi Zhang, Yan Huang, Yi Xu, Peiqing Zhao and Xinxin Xiang analysed and interpreted the data; Yingyi Zhang and Yi Xu collected the serum and tissue samples. Zhe Kong, Xuechao Wan and Yajuan Liu wrote, reviewed and revised the manuscript.

## Data Availability

The data that support the findings of this study are available from the corresponding author upon reasonable request.
