# Supplementary figures and images for: Circular RNA circFOXO3 promotes prostate cancer progression through sponging miR‐29a‐3p
Source: J Cell Mol Med. 2019 Nov 16;24(1):799–813. doi: 10.1111/jcmm.14791 (PMC6933405; doi:10.1111/jcmm.14791)

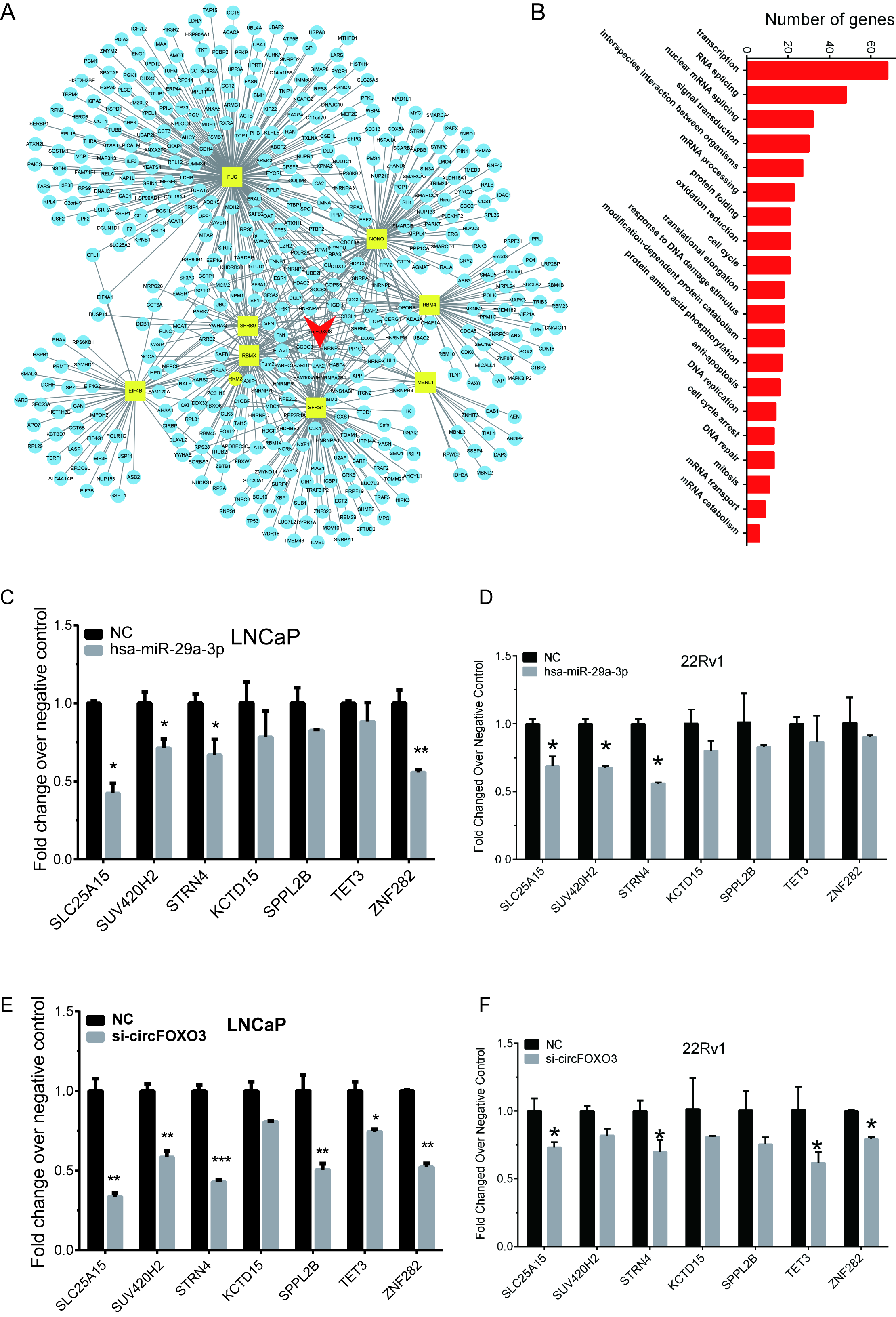

Supplement: Supplementary file 1 [file JCMM-24-799-s001.tif]
